# Umbilical Cord Blood-Derived Stem Cells Improve Heat Tolerance and Hypothalamic Damage in Heat Stressed Mice

**DOI:** 10.1155/2014/685683

**Published:** 2014-04-07

**Authors:** Ling-Shu Tseng, Sheng-Hsien Chen, Mao-Tsun Lin, Ying-Chu Lin

**Affiliations:** ^1^School of Dentistry, Kaohsiung Medical University, Kaohsiung City 807, Taiwan; ^2^Department of Medical Research, Chi Mei Medical Center, Tainan City 710, Taiwan; ^3^Da-An Women and Children Hospital, Tainan City 710, Taiwan; ^4^Department of Biotechnology, Southern Taiwan University of Science and Technology, Tainan City 710, Taiwan

## Abstract

Heatstroke is characterized by excessive hyperthermia associated with systemic inflammatory responses, which leads to multiple organ failure, in which brain disorders predominate. This definition can be almost fulfilled by a mouse model of heatstroke used in the present study. Unanesthetized mice were exposed to whole body heating (41.2°C for 1 hour) and then returned to room temperature (26°C) for recovery. Immediately after termination of whole body heating, heated mice displayed excessive hyperthermia (body core temperature ~42.5°C). Four hours after termination of heat stress, heated mice displayed (i) systemic inflammation; (ii) ischemic, hypoxic, and oxidative damage to the hypothalamus; (iii) hypothalamo-pituitary-adrenocortical axis impairment (reflected by plasma levels of both adrenocorticotrophic-hormone and corticosterone); (iv) decreased fractional survival; and (v) thermoregulatory deficits (e.g., they became hypothermia when they were exposed to room temperature). These heatstroke reactions can be significantly attenuated by human umbilical cord blood-derived CD34^+^ cells therapy. Our data suggest that human umbilical cord blood-derived stem cells therapy may improve outcomes of heatstroke in mice by reducing systemic inflammation as well as hypothalamo-pituitary-adrenocortical axis impairment.

## 1. Introduction

Heat exposure causes an increase in c-fos mRNA and protein in different brain regions including the hypothalamus [1]. Large releases have been reported in brain norepinephrine [2], dopamine, serotonin [3], and glutamate [4]. In addition, heat stress causes the increase in hypothalamic numbers of c-fos-positive cells [5, 6] as well as the increase in blood concentrations of both adrenocorticotrophic-hormone (ACTH) and corticosterone [7, 8], suggesting mobilization of the hypothalamic-pituitary-adrenocortical (HPA) axis. According to the findings of Michel et al. [9], intolerance to heat exposure is associated with HPA axis impairment.

Human umbilical cord blood cells (HUCBC) have emerged as an alternative to bone marrow since they have greater availability, lower risk of mediating viral transmission, and weaker immunogenicity [10]. It has also been documented that transplantation of HUCBC is a promising therapeutic strategy against stroke, traumatic brain injury, spinal cord injury, and heatstroke [11–14]. Although we have demonstrated that HUCBC therapy resuscitates heatstroke rats (under anesthesia) by reducing hypothalamic apoptosis [15], evidence is not available about the protective effect of HUCBC-derived CD34^+^ cells against the heat intolerance, systemic inflammation, HPA axis impairment, and ischemic and oxidation damage to the hypothalamus in unanesthetized mice under heat stress.

To deal with the hypothesis, in the present study, heat tolerance was evaluated by assessing occurrence of thermoregulatory deficit as well as lethality after heat exposure [16, 17]. HPA axis impairment was reflected by the plasma levels of both ACTH and corticosterone in response to heat stress [9]. In addition, hypothalamic levels of cellular ischemia markers (e.g., cerebral blood flow [CBF], glutamate, and lactate/pyruvate ratio), oxidative damage indicators (e.g., malondialdehyde [MDA], oxidative- and reduced- form glutathione [GSH and GSSG], glutathione peroxidase [GP_*x*_], glutathione reductase [GR], nitric oxide [NO_*x*_^−^], and 2,3-dihydroxybenzic acid [2,3-DHBA]), and plasma levels of inflammatory indicators (e.g., tumor necrosis factor-*α* [TNF-*α*], interleukin-10 [IL-10], and ICAM-1) in heat stressed mice treated with CD34^+^ cells or vehicle were assessed [18].

## 2. Material and Methods

### 2.1. Human CD34^+^ Cell Preparation

Human CD34^+^ cells were isolated from the cord blood of 15 females after informed consent from the mother and IRB approval. Single-cell suspensions of 1 × 10^5^/0.3 mL of HUCBC were administered via the tail vein immediately after the termination of whole body heating (WBH). All protocols were approved by the Animal Ethics Committee of the Chi Mei Medical Center (Tainan, Taiwan) in accordance with the Guide for the Care and Use of Laboratory Animals of the National Science Council and the Guidelines of the Animal Welfare Act.

Human CD34^+^ cells were isolated from cord blood using a Direct CD34^+^ Progenitor Cell Isolation kit (Miltenyi Biotec, Bergisch Gladack, Germany) and CD34^+^ Multisort kit (Miltenyi Biotec) according to the manufacturer's protocol. In brief, human cord blood lymphocytes and monocytes were suspended in 300 *μ*L of phosphate buffered saline (PBS) and 5 mM EDTA. These cells were labeled with a hapten-conjugated monoclonal antibody against CD34 (PharMingen, San Diego, CA), followed by an antihapten antibody coupled with microbeads, and were incubated with beads at ratios of 100 *μ*L of beads per 10^8^ cells for 15 min at 4°C. FACS analysis using anti-CD34 antibodies (Phar Mingen) labeled with phycoerythrin (Becton Dickinson, Mountain View, CA) of MACS-sorted cells showed that 96 ± 3% of the selected cells were positive for CD34.

### 2.2. Murine Model of Heatstroke

ICR (Institute of Cancer Research) of the National Institutes of Health in the USA mice were purchased from the National Animal Center (Taipei, Taiwan) and kept under a 12 h light-dark cycle at controlled temperature (22 ± 2°C) with free access to food and tap water: ICR male mice 8- to 10- week-old were exposed to WBH (41.2°C, relative humidity 50%–55%, and for 1 hour) in an environmental temperature-controlled chamber [16, 17, 19]. The heated mice were returned to the normal room temperature (26°C) after the end of WBH. Mice that survived to day 4 of WBH were considered survivals, and the data were used for analysis of the results. In separate experiments, 4 h following WBH, all of the animals were killed and their organs were removed for histological and biochemical evaluation. The contents of NO_*x*_^−^, 2,3-DHBA, glutamate, lactate-to-pyruvate ratio, glycerol, MDA, GSSG, GSH, GP_*x*_ and GR, and number of neuronal damage scores in the hypothalamus were determined. In addition, systemic inflammatory responses molecules in the peripheral blood stream were assessed. For rectal temperature measurements, unrestrained, unanesthetized mice were used and measurements were collapsed into 10 min averages, taking one mouse each form each group and changing the sequence thereafter. Rectal temperatures were measured by a thermocouple probe (Bailey Instruments, Saddlebrook, NJ, USA).

### 2.3. Assessment of Thermoregulatory Function

Immediately after the termination of WBH, the animals were returned to a room temperature of 26°C for recovery. According to the findings of Chatterjee et al. [16, 17], WBH-treated mice became hypothermia, when they were exposed to room temperature (24°C).

### 2.4. Neuronal Damage Score

At the end of the experiments, animals were killed by an overdose of sodium pentobarbital, and the brains were fixed in situ and left in skull in 100% neutral-buffered formalin for at least 24 hours before removal from the skull. The brain was removed and embedded in paraffin blocks. Serial sections (10 *μ*m thick) through the hypothalamus were stained with hematoxylin and eosin for microscopic examination. The extent of neuronal damage was scored on a scale of 1 to 3, modified from the grading system of Pulsinelli et al. [20], in which 0 is normal, 1 indicates approximately 30% of the neurons are damaged, 2 indicates that approximately 60% of the neurons are damaged, and 3 indicates that 100% of the neurons are damaged. Each hemisphere was evaluated independently by an examiner blinded to the experimental conditions.

### 2.5. Assessment of CBF and Cerebral PO_**2**_

A 100 *μ*m diameter thermocouple and two 230 *μ*m fibers were attached to the oxygen probe. This combined probe measured oxygen, temperature, and microvascular blood flow. These measurements required OxyLite and Oxyflo instruments. OxyLite 2000 (Oxford Optronix Ltd., Oxford, UK) is a 2-channel device (measuring PO_2_ and temperature at two sites simultaneously), whereas OxyFlo 2000 is a 2-channel laser doppler perfusion monitoring instrument. Under anesthesia, the mouse was placed in a stereotaxic apparatus and the combined probe was implanted in to the brain (or the hypothalamus) using the atlas and coordinates of Paxinos and Watson [21].

### 2.6. Extracellular Levels of Glutamate, Lactate-to-Pyruvate Ratio, Glycerol, NO_*x*_^−^, and 2,3-DHBA in the Hypothalamus

Hypothalamic samples were homogenized in 0.05 M phosphate buffer, pH7.0 and then centrifuged at 4000 ×g for 20 min at 4°C. The supernatants were used for determination of cellular levels of glutamate, lactate-to-pyruvate ratio, glycerol, NO_*x*_^−^, and 2,3-DHBA. The dialysis probe (4 mm in length CMA/12; Carnegie Medicine, Stockholm, Sweden) was put into the supernatants to obtain the dialysates.

The nitric oxide (NO_*x*_^−^) concentration in the dialysates of hypothalamus was measured with the Eicom ENO-20 NO_*x*_^−^ analysis system [22]. In the Eicom ENO-20 NO_*x*_^−^ analysis system, after the NO_2_^−^ and NO_3_^−^ in the sample have been separated by the column, the NO_2_^−^ reacts in the acidic solution with the primary aromatic amine to produce an azo compound. Following this, the addition of aromatic amines to the azo compound results in a coupling that produces a diazo compound and the absorbance rate of the red color in this compound is then measured. For measurement of glutamate, lactate-to-pyruvate ratio, and glycerol, the dialysates were injected into a CMA600 microdialysis analyzer (Carnegie Medicine, Stockholm, Sweden). The concentrations of hydroxyl radicals were measured by a modified procedure based on the hydroxylation of sodium salicylates by hydroxyl radicals, leading to the production of 2,3-dihydroxybenzoic acid and 2,5-dihydroxybenzoic acid [12].

### 2.7. Determination of Lipid Peroxidation

Lipid peroxidation was assessed by measuring the levels of MDA with 2-thiobarbituric acid (TBA) to form a chromophore absorbing at 532 nm [23]. About 0.1 g of tissue was homogenized with 1.5 mL of 0.1 M phosphate buffer at pH3.5. The reaction mixture (0.2 mL of sample, 1.5 mL of 20% acetic acid, 0.2 mL of 8.1% sodium dodecyl sulfate, and 1.5 mL of aqueous solution of 0.8% TBA, up to 4 mL with distilled water) was heated to 95°C for 1 h, and then 5 mL of N-butanol and pyridine (15 : 1 vol/vol) was added. The mixture was vortexed vigorously, centrifuged at 1500 g for 10 min, and the absorbance of the organic phase was measured at 532 nm. The values were expressed as nanomoles of TBA-reactive substances (MDA equivalent) per milligram of protein.

### 2.8. Quantification of Total and Oxidized Glutathione

Tissues were homogenized in 5% 5-eslfoslicylic acid (1 : 10 wt/vol) at 0°C, and the supernatants were used for analysis of total and oxidized glutathione. Total glutathione [reduced-form glutathione (GSH) + oxidized-form glutathione (GSSG)] was analyzed according to the Tieze method [24], and GSSG was determined as described by Griffith [25]. The recycling assay for total glutathione is oxidized by 5,5-Dithiosis [2 acid] (DTNB) to give GSSG with stoichiometric formation of 5-thio-2-nitrobenzoic acid. GSSG is reduced to GSH by the action of the highly specific glutathione reductase (GR) and nicotinamide adenine dinucleotide phosphate (reduced form; NADPH). The rate of 5-thio-2-nitrobenzoic acid formation is followed at 412 nm and is proportional to the sum of GSH and GSSG present.

### 2.9. Determination of Glutathione Peroxidase (GP_*x*_) and Glutathione Reductase (GR) Activity

Tissues were homogenized in 0.05 M phosphate buffer, pH7.0 and then centrifuged at 4000 ×g for 20 min at 4°C. The supernatants were used for GP_*x*_ and GR activity assay. The GP_*x*_ and GR activities were assayed with a commercial GP_*x*_ assay kit (Sigma, USA) and a GP assay kit (Sigma, USA), respectively. One unit of GP_*x*_ and GR activity was defined as the amount of sample required to oxidize 1 mmol of NADPH per minute based on the molecular absorbance of 6.22 × 10^6^ for NADPH.

### 2.10. Plasma Concentrations of Inflammatory and Intracellular Adhesion Molecules and Cytokines

Blood samples were taken at 4 hours after the start of heat exposure for determination of TNF-*α*, IL-10, and intercellular adhesion molecule^−1^ (ICAM-1) levels. The amounts of the cytokines in serum were determined by double antibody sandwich enzyme-linked immunosorbent assay (R&D systems, Minneapolis, MN) according to the manufacturer's instructions. Optical densities were read on a plate reader set at 450 nm for TNF-*α*, IL-10, and ICAM-1. The concentration of TNF-*α*, IL-10, and ICAM-1 in the serum samples was calculated from the standard curve multiplied by the dilution factor and was expressed as picograms per milliliter [15].

### 2.11. Plasma Assessment of ACTH and Corticosterone

Plasma ACTH and corticosterone were assayed using ACTH (Rat, Mouse)-RIA kit (Phoenix Pharmaceuticals, Burlingame, CA, USA) and Corticosterone Double Antibody RIA kit (MP Biomedicals, Solon, OH, USA), respectively. All analyses were performed according to manufacturers' instructions.

### 2.12. Statistical Analysis

All values in the figures and text are expressed as mean ± S.E.M. of n observations, where n represents the number of animals studied. Statistical evaluation was performed by using analysis of variance (ANOVA) followed by a multiple-comparison test (Scheffe's test). The Kaplan Meier analysis was used for determining the significant differences in the survival rate between control and drug-treated groups. The Wilcoxon tests were used for evaluation of neuronal damage scores. The Wilcoxon test converts the scores or values of a variable to ranks, requires calculation of a sum of the ranks, and provides critical values for the sum necessary to test the null hypothesis at a given significant levels. These data were presented as “median”, followed by first (Q1) and third (Q3) quartile. A *P* value of less than 0.05 was considered to be statistically significant.

## 3. Results

### 3.1. Thermoregulatory Outcome and Lethality Induced by WBH

**Figure 1:**
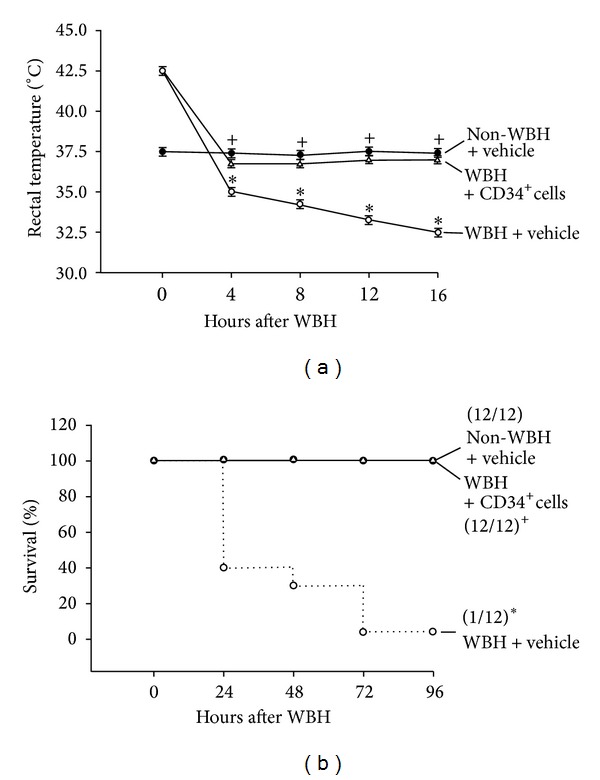
Thermoregulatory deficits and lethality by WBH. (a) Thermoregulatory dysfunction (or animals become hypothermia when exposing them to room temperature, 26°C) caused by whole body heating (WBH; 41.2°C for 1 h). Data are expressed as means ± S.E.M. of 12 mice per group. *At time “0” h after WBH (or immediately after the termination of WBH), heated mice treated with vehicle (WBH + vehicle; “○”) or heated mice treated with CD34^+^ cells (WBH + CD34^+^ cells; “△”) had significantly higher rectal temperature (~42.5°C) than those of non-WBH mice treated with vehicle (non-WBH + vehicle; “●”). In contrast, at time “4” h, “8” h, “12” h, or “16” h after WBH, (WBH + vehicle) group had significantly lower rectal temperature (Tco; ~35°C–~32.5°C) than those of (non-WBH + vehicle) group. ^+^At time “4” h, “8” h, “12” h, or “16” h after WBH, (WBH + CD34^+^ cells) group had significantly higher rectal temperature than those of (WBH + vehicle) group (*P* < 0.01). (b) Lethality (or decreased percentage survival) caused by WBH. *P < 0.01, (non-WBH + vehicle) mice versus (WBH + vehicle) mice. ^+^ *P* < 0.01, (WBH + CD34^+^ cells) group versus (WBH + vehicle) group.

Functional tests showed balanced thermoregulatory deficits between vehicle-treated WBH group and CD34^+^ cells-treated WBH mice. CD34^+^ cells-treated WBH mice showed significant (*P* < 0.05, *n* = 12/group) improvement of functional recovery on thermoregulatory test at 4–16 h compared with vehicle-treated WBH mice (Figure 1). The survival of CD34^+^-treated WBH mice was 12 of 12 mice and one of 12 for vehicle-treated WBH mice (Figure 1).

### 3.2. Hypothalamic Cells Damage Induced by WBH

**Figure 2:**
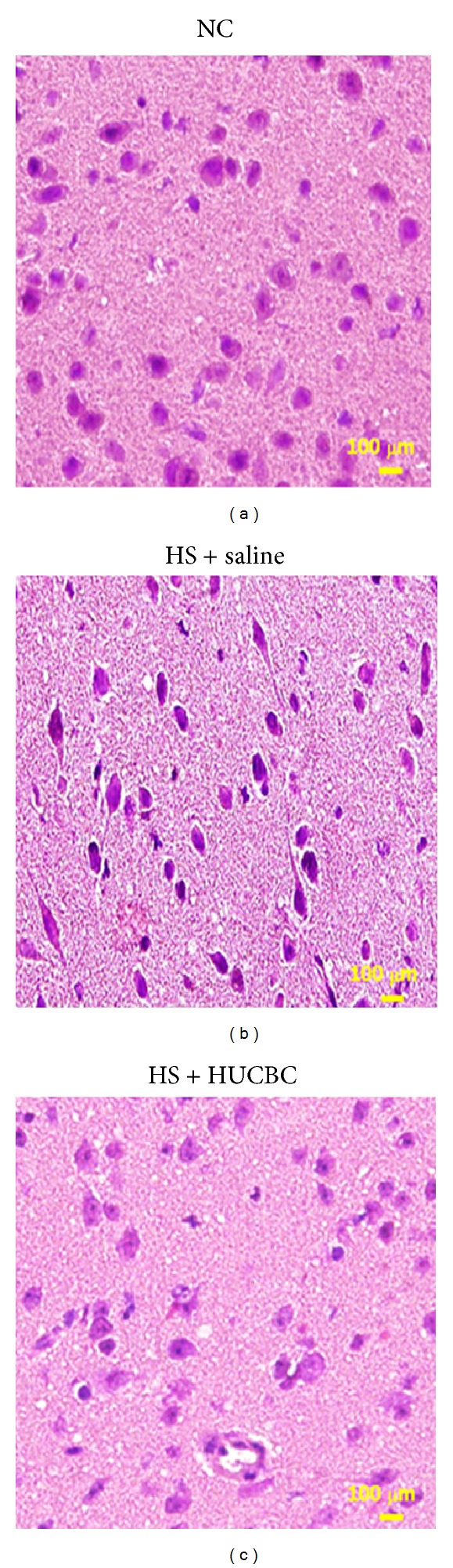
Hypothalamic hematoxylin-eosin (HE) staining 4 hours after WBH. Photographs showing hypothalamic HE staining for a (non-WBH + vehicle), a (WBH + vehicle) mouse, and a (WBH + CD34^+^ cells) mouse. Samples were obtained 4 hours after WBH for the WBH groups or the equivalent time period for the non-WBH group. (WBH + vehicle) mice showed cell body shrinkage, pyknosis of the nucleus, loss of Nissl substance, and disappearance of the nucleolus which could be attenuated by CD34^+^ cells treatment.

Histological verification showed that hypothalamic values of cell damage score (Table 1, Figure 2) for vehicle-treated WBH mice were significantly higher 4 h after WBH than they were for the non-WBH control mice. Vehicle-treated WBH mice displayed cell body shrinkage, pyknosis of the nucleus, loss of Nissl substance, and disappearance of the nucleolus (Figure 2). As compared to non-WBH control mice, vehicle-treated WBH mice also had significantly higher levels of a cellular damage marker (e.g., glycerol) (Table 2) in the hypothalamus [26, 27]. CD34^+^ cells-treated WBH mice showed significant improvement of hypothalamic cell damage (Tables 1 and 2).

### 3.3. Hypothalamic Ischemia and Hypoxia Induced by WBH

Intracerebral assessment revealed that hypothalamic levels of both CBF and PO2 for vehicle-treated heated mice were significantly lower at 4 h after WBH than they were for the non-WBH mice (Table 2). Again, the hypothalamic levels of cellular ischemia markers (e.g., glutamate and lactate/pyruvate ratio) [26, 27] for vehicle-treated WBH mice were significantly higher at 4 h after WBH than they were for the non-WBH control mice (Table 2). WBH mice treated with CD34^+^ cells showed significant improvement of cerebral ischemia and hypoxia (Table 2).

### 3.4. Oxidative Stress Induced by WBH

Biochemical determination showed that hypothalamic levels of MDA, GSSG/GSH, NO_*x*_^−^, and 2,3-DHBA for vehicle-treated WBH mice were all significantly higher at 4 h after WBH than they were for the non-WBH control mice (Table 3). On the other hand, hypothalamic levels of GP_*x*_ and GR for vehicle-treated WBH mice were significantly lower than they were for the non-WBH control mice (Table 3). WBH mice treated with CD34^+^ cells showed significant improvement of oxidative stress caused by WBH (Table 3).

### 3.5. Increased Plasma Levels of both ACTH and Corticosterone Induced by WBH

Biochemical verification showed that plasma levels of both ACTH and corticosterone for vehicle-treated WBH mice were significantly higher 4 h after WBH than they were for the non-WBH control mice (Table 4). WBH mice treated with CD34^+^ cells showed significant enhancement of both ACTH and corticosterone in plasma by WBH (Table 4).

### 3.6. Increased Serum Levels of Systemic Inflammatory Response Indicator Induced by WBH

Biochemical determination showed that serum levels of several systemic inflammatory response indicators such as TNF-*α* and ICAM-1 for vehicle-treated heated mice were significantly higher 4 h after WBH than they were for the non-WBH mice (Table 4). WBH mice treated with CD34^+^ cells showed significant reduction of the increased serum levels of these 2 inflammatory response indicators by WBH (Table 4). Table 4 also demonstrated that the serum levels of an anti-inflammatory cytokine, IL-10, were further significantly increased following CD34^+^ cells therapy.

## 4. Discussion

Heat tolerance varies considerably among individuals [28]. When exposed to a certain extent of heat exposure, some subjects display a slightly elevated body core temperature (Tco < 40°C) while others become severely ill with a Tco above 40°C. When rats are exposed to heat, they also show a wide interindividual variability [9]. Heat tolerant rats showing the lowest Tco had a highest plasma ACTH and corticosterone levels. Conversely, heat intolerant rats exhibiting the highest Tco had the lowest plasma ACTH and corticosterone. These investigators also provide data to promote that decreased heat tolerance is associated with HPA axis impairment in rats. Consisting with the above hypothesis, we showed that vehicle-treated WBH mice exhibiting lowest survival showed the lowest plasma ACTH and corticosterone levels. In contrast, CD34^+^ cells-treated WBH mice presented a greater percentage survival as well as a greater plasma level of both ACTH and corticosterone. The mobilization of HPA axis activity is associated with the increase in blood ACTH and corticosterone concentrations [7, 8]. In addition, our previous results have shown that corticosterone supplementation has beneficial effects in treating heatstroke in rats [28]. It is likely that CD34+ cells therapy may improve heat tolerance by attenuating HPA axis impairment in mice during heat exposure.

Accumulating evidence has demonstrated that CD34^+^ cells transplantation is a promising therapeutic method against cerebral ischemic diseases, such as stroke, traumatic brain injury, and spinal cord injury [11, 13, 14]. Our previous [12] and present results have also shown that HUCBC-derived CD34^+^ cells therapy has beneficial effects in heatstroke. Severe heat stress decreases mean arterial pressure (MAP), increases intracranial pressure (ICP), and results in decreased cerebral perfusion pressure (CPP = MAP − ICP), which leads to cerebral ischemia and hypoxia [18]. In addition, hypothalamic and plasma values of cellular ischemia and damage markers, prooxidant enzymes, proinflammatory cytokines, inducible nitric oxide synthase-dependent nitric oxide, and myeloperoxidase activity were all significantly elevated after heatstroke occurrence [18]. 

In particular, heatstroke causes overproduction of proinflammatory cytokines in both the brain and the peripheral blood stream; this is associated with decreased MAP. In fact, activated inflammation is involved in the severity of acute heart failure [29], septic shock [30], and circulatory shock [31]. Systemic administration of interleukin-1 receptor [32] or glucocorticoids [28] immediately after the onset of heatstroke is able to attenuate arterial hypotension and cerebral ischemia and injury and to improve survival. In an anesthetized rat model of heatstroke, CD34^+^ cell therapy significantly attenuates arterial hypotension, intracranial hypertension, cerebral ischemia, hypoxia, and injury, and TNF-*α* overproduction during heatstroke [33]. In order to avoid the influence of anesthetic state, our data further demonstrate that in an unanesthetized mouse model of heatstroke, CD34^+^ cells therapy promotes survival by attenuating overproduction of systemic inflammatory response molecules (e.g., TNF-*α* and ICAM-1) and ischemic, hypoxic, and oxidative damage to the hypothalamus. In fact, both anti-inflammatory and proinflammatory cytokines normally have a role to fight infection and prevent immune pathology, respectively [34]. Interleukin-10 has important anti-inflammatory and immunosuppressive properties through attenuation of TNF-*α* and other proinflammatory cytokines [31]. Thus, it appears that CD34^+^ therapy may improve brain inflammation during heatstroke by stimulating production of IL-10.

An occurrence of local inflammation process may be considered since TNF-*α* mRNA decreased in the tolerant rats' hypothalamus and pituitary as compared with control rats [9]. On the contrary, the occurrence of higher stimulation by free radicals led to an increase in the TNF-*α* mRNA level in the heat exhausted rats. [9, 35]. Heat tolerant rats exhibit low IL-1*β* and TNF-*α* mRNAs as well as high corticosterone levels, whereas heat exhausted rats present high IL-1*β* and TNF-*α* mRNA, but low corticosterone level [9]. As shown in Figure 1, animals displayed hyperthermia immediately after termination of WBH. Four hours after WBH, vehicle-treated WBH mice showed activated inflammation, hypothalamic ischemia, and HPA axis impairment, which could be significantly attenuated by CD34^+^ cells therapy. 

As compared to heated mice treated with vehicle solution, heated mice treated with CD34^+^ cells displayed lower hypothalamic values of cellular ischemia (e.g., glutamate and lactate-to-pyruvate ratio), damage (e.g., glycerol) markers, and prooxidant enzymes (e.g., lipid peroxidation and glutathione oxidation). In contrast, CD34+ cells-treated heated mice had higher hypothalamic values of antioxidant defences (e.g., glutathione peroxidase and glutathione reductase). These observations suggest that heat-induced oxidative damage to hypothalamus in mice can be attenuate by CD34^+^ cells therapy.

## 5. Conclusion

Heatstroke is characterized by excessive hyperthermia associated with systemic inflammatory responses, which leads to multiple organ failure, in which brain disorders predominates. This definition can be almost fulfilled by our present animal model [26, 36]. Heatstroke mice displayed (i) systemic inflammation; (ii) ischemic, hypoxic, and oxidative damage to the hypothalamus; (iii) hypothalamic-pituitary-adrenocortical axis impairment; (iv) decreased survival; and (v) thermoregulatory deficit. These heatstroke reactions can be significantly attenuated by HUCBC-derived CD34^+^ cells therapy. Our data suggest that CD34^+^ cells therapy may improve heat tolerance by reducing systemic inflammation and HPA axis impairment in heatstroke mice.

## Figures and Tables

**Table 1:** Effects of heat exposure on neuronal damage score of the brain (or the hypothalamus) in different groups of mice.

Treatment groups	Neuronal damage score (0–3)
(1) Non-WBH mice	0 (0, 0)
(2) Non-WBH mice treated with CD34^+^ cells (1 × 10^5^ cells/0.3 mL, i.v.)	0 (0, 0)
(3) Heated mice treated with vehicle saline (0.3 mL, i.v.)	2 (2, 2)^a^
(4) Heated mice treated with CD34^+^ cells (1 × 10^5^ cells/0.3 mL, i.v.)	0.75 (0, 0.75)^b^

Samples were measured 4 hours after whole body heating (WBH; 41.2°C for 1 houer) or the equivalent time period for non-heated group. ^a^compared with non-WBH group (*P* < 0.01); ^b^compared with group 2 (*P* < 0.05). Data are means ± S.E.M. of 12 mice per group.

**Table 2:** Effects of heat exposure on hypothalamic levels of glutamate, lactate/pyruvate, glycerol, cerebral blood flow (CBF), and PO_2_ in different groups of mice.

Treatment groups	Glutamate (percentage of baseline)	Lactate/pyruvate ratio	Glycerol (percentage of baseline)	CBF (BPU)	PO_2_ (mmHg)
(1) Non-WBH mice	98 ± 5	10 ± 4	99 ± 6	328 ± 23	21 ± 2
(2) Non-WBH mice treated with CD34^+^ cells (1 × 10^5^ cells/0.3 mL, i.v.)	100 ± 6	12 ± 5	98 ± 7	307 ± 24	21 ± 3
(3) Heated mice treated with vehicle saline (0.3 mL, i.v.)	196 ± 22^a^	231 ± 32^a^	198 ± 16^a^	162 ± 11^a^	10 ± 1^a^
(4) Heated mice treated with CD34^+^ cells (1 × 10^5^ cells/0.3 mL, i.v.)	142 ± 10^b^	77 ± 11^b^	66 ± 12^b^	245 ± 16^b^	16 ± 2^b^

Samples were measured 4 hours after whole body heating (WBH) or the equivalent time period for non-heated group. ^a^compared with non-WBH group (*P* < 0.01); ^b^compared with group 2 (*P* < 0.05). Data are means ± S.E.M. of 12 mice per group.

**Table 3:** Effect of heat exposure on hypothalamic levels of malondialdehyde (MDA), oxidative-form glutathione (GSSG)/reduced-form glutathione (GSH), glutathione peroxidase (GP_*x*_), glutathione reductase (GR), nitric oxide metabolites (NO_*x*_^−^), and 2,3-dihydroxy benzoic acid (2,3-DHBA) in different groups of mice.

Treatment groups	MAD (nmol/mg protein)	GSSG/GSH	GP (mU/mg protein)	GR (mu/mg protein)	NO_*x*_^−^ (*µ*M)	2,3-DHBA (percentage of baseline)
(1) Non-WBH mice	5 ± 2	0.45 ± 0.14	314 ± 36	175 ± 16	19 ± 2	100 ± 6
(2) Non-WBH mice treated with CD34^+^ cells	4 ± 2	0.42 ± 0.16	302 ± 33	169 ± 17	17 ± 3	99 ± 5
(3) WBH mice treated with vehicle saline	12 ± 2^a^	2.43 ± 0.38^a^	83 ± 17^a^	81 ± 13^a^	115 ± 12^a^	188 ± 10^a^
(4) WBH mice treated with CD34^+^ cells	4 ± 2^b^	0.42 ± 0.15^b^	257 ± 28^b^	166 ± 15^b^	21 ± 4	103 ± 5^b^

Samples were measured 4 hours after whole body heating (WBH; 41.2°C for 1 hour) or the equivalent period for non-WBH. ^a^compared with non-WBH group (*P* < 0.01); ^b^compared with group 2 (*P* < 0.05). Data are means ± S.E.M. of 12 mice per group.

**Table 4:** Effect of heat exposure on plasma levels of adrenocorticotrophic hormone (ACTH), corticosterone, tumor necrosis factor-*α* (TNF-*α*), interleukin-10 (IL-10), and ICAM-1 for various groups of mice.

Treatment groups	ACTH (pg·mL^−1^)	Corticosterone (ng·mL^−1^)	TNF (pg/mL)	IL-10 (pg/mL)	ICAM (pg/mL)
(1) Non-WBH mice	372 ± 79	32 ± 19	10 ± 6	5 ± 3	9 ± 4
(2) Non-WBH mice treated with CD34^+^ cells	361 ± 72	29 ± 17	8 ± 5	6 ± 2	11 ± 5
(3) WBH mice treated with saline	601 ± 98^a^	256 ± 25^a^	415 ± 82^a^	11 ± 4^a^	496 ± 22^a^
(4) WBH mice treated with CD34^+^ cells	1764 ± 116^b^	643 ± 30^b^	37 ± 6^b^	83 ± 11^b^	67 ± 18^b^

Samples were measured 4 hours after whole body temperature (WBH; 41.2°C for 1 hour) or the equivalent time period for non-WBH group. ^a^compared with non-WBH group (*P* < 0.01); ^b^compared with group 2 (*P* < 0.01). Data are means ± S.E.M. of 12 mice per group.

## Abbreviations

WBH: Whole body heating
HUCBC: Hypothalamic-pituitary-adrenal
ACTH: Adrenocorticotropic hormone
NO_*x*_^−^: Nitric oxide metabolites
2,3-DHBA: 2,3-dihydroxybenzoic acid
GSSG: Oxidative-form glutathione
GSH: Reduced-form glutathione
GP_*x*_: Glutathione peroxidase
GR: Glutathione reductase
CBF: Cerebral blood flow
ICAM-1: Intercellular adhesion molecule-1
MDA: Malondialdehyde
TBA: 2-Thiobarbituric acid
TNF-*α*: Tumor necrosis factor-alpha
IL-10: Interleukin-10
IL-1*β*: Interleukin-1*β*.
